# T1/T2-weighted ratio is a surrogate marker of demyelination in multiple sclerosis: No

**DOI:** 10.1177/13524585211063622

**Published:** 2022-01-22

**Authors:** Mark Mühlau

**Affiliations:** Department of Neurology, School of Medicine, Technical University of Munich, Munich, Germany/TUM-Neuroimaging Center, School of Medicine, Technical University of Munich, Munich, Germany

In the last decade, ratio maps derived from T1-weighted (T1w) and T2-weighted (T2w) magnetic resonance imaging (MRI) sequences have been increasingly used particularly to investigate the cerebral cortex. The belief that this measure primarily reflects myelin together with the possibility that it can be generated from two conventional MRI sequences – even in retrospect – certainly makes it very attractive for multiple sclerosis (MS) research. When discussing whether T1w/T2w ratio maps should be referred to as ‘myelin maps’, it is essential to recapitulate the basic idea of T1w/T2w ratio imaging and to distinguish between brain white and grey matter.

To put a damper on high expectations first, we should recognize that T1w/T2w ratio maps can at its best be a rough estimate of myelin with limited specificity, since the short T2 times (<<1 ms) of macromolecular protons abundant in the lipids and proteins that constitute myelin are not precisely covered by conventional sequences. Furthermore, conventional sequences cannot easily be calibrated, because of the instability of the magnetic field in MRI scanners, which hampers intensity quantification. Intriguingly, it has been hypothesized that T1w and T2w sequences are affected by these instabilities to a similar degree (when acquired in close temporal proximity), allowing for some quantification of intensity through dividing the T1w by the T2w intensity (when using identical sequence parameters at the same scanner for all subjects under investigation).^1^ Thus, the original concept does not emphasize the ratio but the scaling of T1w intensities, leading to the question to what extent the T1w signal reflects myelin.

For brain white matter, this assumption is straightforward and plausible for several reasons. The mass fraction of myelin in brain white matter is considerable and myelin consists of about 80% lipids, which have a high T1w signal. Hence, it seems plausible that myelin considerably drives the T1w signal of brain white matter. Moreover, myelin maturation during brain development is perfectly paralleled by the T1w signal change from hypo- to hyperintense in humans, which has been confirmed histologically in pigs.

In contrast, the assumption that myelin content can be estimated by the T1w/T2w ratio also in brain grey matter lacks plausibility. For deep grey matter, interference with iron content, decreasing the T2w signal disproportionally, is very likely. The application of the concept to the cerebral cortex has become most popular. However, the myelin content of the cerebral cortex is far below that of white matter^2^ although the T1w signal intensity is about half of that of white matter. It should also be kept in mind that alternative drivers of the T1w signal are well conceivable given that numerous tissues, containing virtually no myelin, have similarly high T1w signal intensities (e.g. the temporal muscle in a T1w brain image). Surprisingly, the interpretation of T1w/T2w cortical maps as ‘myelin maps’ has been brought forward in high-ranked publications but has been substantiated neither by quantitative data nor by statistical means. The most popular argument favouring the idea of T1w/T2w cortical maps as ‘myelin maps’^1^ is that MRI-based T1w/T2w ratio maps resemble early histology-based myelin maps by Hopf. Although these comparisons have been performed intensively^3^ and a new version of the myelin maps by Hopf has been rendered, evidence has remained indirect and interpretation of results has become circular (i.e. other measures have been related to T1w/T2w cortical maps but interpreted in relation to ‘myelin’).^3^ Of note, studies in the field of MS did relate T1w/T2w maps with histological data; their results do not lend support to the concept. Although within demyelinated cortical lesions, the intensity of the T1w/T2w ratio was demonstrated to be decreased in an early study,^4^ a later study concluded that cortical changes other than demyelination influence the T1w/T2w signal.^5^ When comparing T1w/T2w values between normal-appearing and lesioned tissue in both brain grey and white matter at 7 Tesla, a striking difference was indeed found in white matter, while a difference in grey matter between lesioned and non-lesioned tissue was hardly detectable (i.e. small when compared to control regions and non-significant when compared to whole brain normal-appearing grey matter of controls).^6^ Our own study related several histology-based measures from nine postmortem cases to T1w/T2w ratio values across cortical regions, defined by a standard atlas, and found a stronger correlation with synaptic density than with myelin – a finding certainly in need of replication but neither in support of the T1w/T2w ratio representing myelin.^7^

Should we continue with T1w/T2w cortical maps? Yes! There are hardly alternative options to extract (semi)quantitative parameters from conventional MRI (beyond volumes) and exciting associations with cognitive function in health^8^ and MS^7,9^ have been reported suggesting that this signal may be biologically meaningful. However, methodological issues need to be addressed. How should volumetric effects, particularly in the cortex, be prevented? The essential assumption of similar magnetic field fluctuations of T1w and T2w sequences is unlikely to be valid for all scanners.^10^ In consequence, there is no common agreement whether and how to normalize T1w and T2w image intensities before dividing the former by the latter or whether division by the T2w signal is expedient in combination with other processing steps at all.

In conclusion, T1w/T2w maps are of potential value also for MS research, but we should stop referring to cortical T1w/T2w maps as ‘myelin maps’ insinuating the false impression of compelling evidence on its biological underpinning.
